# Intimate relationships and hypnosis: insecure adult attachment affects emotions and absorption during hypnosis

**DOI:** 10.3389/fpsyg.2024.1326170

**Published:** 2024-02-15

**Authors:** Gloria Di Filippo, Rinaldo Livio Perri

**Affiliations:** ^1^Department of Psychological, Economic and Communication Sciences, University Niccolò Cusano, Rome, Italy; ^2^De Sanctis Clinical Center (CCDS), Rome, Italy

**Keywords:** hypnosis, adult attachment, absorption, consciousness, hypnotizability

## Abstract

**Introduction:**

Hypnosis research indicates that subjects are not equally hypnotizable. Most studies on hypnotizability focused on the relationships with personality or cognitive variables. At the same time, only a few proposed the contribution of the attachment style, defined as the result of the childhood relationship with the caregivers and influencing the adult relations.

**Methods:**

In the present investigation, two studies were carried out to test the possible association between adult attachment and hypnotic responsivity. The adult attachment was assessed using the Experiences in Close Relationships-Revised (ECR-R) questionnaire, while hypnosis was assessed through the Harvard Group Scale of Hypnotic Susceptibility (HGSHS:A; Study 1) and the Phenomenology of Consciousness Inventory: Hypnotic Assessment Procedure (PCI-HAP; Study 2) in order to adopt a behavioral and a phenomenological approach, respectively.

**Results:**

Analyses showed that attachment factors (anxiety and avoidance) were not associated with the level of hypnotizability, whereas it was associated with variations of consciousness during hypnosis, mainly internal dialogue, absorption and negative emotions. Overall, the insecure attachment styles yielded increased mind wandering and restlessness during hypnosis when compared to the secure style. The reason probably lies in the feeling of anxiety or danger of insecurely attached individuals when involved in intimate or confidential relationships.

**Conclusion:**

These findings clarify a still poorly investigated aspect concerning the influence of attachment style on hypnotic experience and further highlight the need to consider inter-individual differences and the phenomenological perspective when assessing hypnosis and hypnotizability.

## 1 Introduction

Hypnotizability refers to “An individual’s ability to experience suggested alterations in physiology, sensations, emotions, thoughts, or behavior during hypnosis” (Elkins et al., 2015). In experimental hypnosis, it is fundamental to quantify hypnotizability that however is often measured in terms of behavioral response to suggestions. This approach mainly detects the construct of suggestibility which does not necessarily reflect the hypnosis experience (Jensen et al., 2017) making still complex a shared definition of hypnotizability (Perri, 2022). For decades, behavioral methods have been successfully used to assess hypnotic responsiveness, however, a growing interest in subjective measures has been developing in recent years (Forbes and Pekala, 1993; Lush et al., 2018; Facco, 2022; Kekecs et al., 2022; Perri, 2022). On the contrary, the main risk of a purely behavioral investigation could be to neglect the phenomenological variations of consciousness featuring the hypnosis experience (Pekala et al., 2010a). The studies concerning non-ordinary states of consciousness rely on the reflexive capacity of the subjects and its modulation and cannot therefore disregard the use of first-person methods to investigate the experiential process (Timmermann et al., 2023).

Most of the studies on hypnotizability focused mainly on its relationship with personality (Barber, 1964; Zhang et al., 2017) cognitive (Raz, 2005; Hiltunen, 2022) or neurophysiological factors (for a review see Landry et al., 2017). A few studies have also investigated the contribution of the adult attachment on hypnotizability (Peter et al., 2011, 2014), with the anxious attachment influencing the relationship between hypnotizability and dissociation (Wieder and Terhune, 2019).

Attachment was defined by Bowlby as the relational model resulting from early relational experiences with caregivers (Bowlby, 1988) and influencing the internal operating models, i.e., the set of schema mediating interpretation of events and influencing how individuals deal with intimate relationships. Attachment is typically classified into four styles: secure attachment, avoidant, anxious, and preoccupied (the last three classified as insecure attachment styles) (Bartholomew and Horowitz, 1991). These styles reflect various shades of intimacy avoidance and needs for acceptance from others to maintain positive self-esteem. For example, individuals with a preoccupied attachment style may seek closeness with others to satisfy their need for dependence, while those with an anxious style may avoid involvement for fear of disappointment. Because of the key role of expectation and relationships in hypnosis (see Kirsch, 2000) it is possible to hypothesize a contribution of the attachment style on the ability to experience hypnosis and, to a larger extent, on hypnotizability. In other words, the influence the early parent–child relational patterns may have on the hypnotized-hypnotist relationship could be relevant to the perceived hypnotic experience (Varga, 2021). In fact, securely attached individuals are more likely to report pleasant feelings in therapy (Obegi and Berant, 2010) as they learned the functional value and safety of relationships (Wallin, 2007); also, they share with the high hypnotizables the greater ability to explore their inner world (Facco et al., 2017). On the contrary, individuals who experienced repeated failures in the caregiving process may be more prone to avoid relationships, feeling uncomfortable in intimate or confidential relationships, and experiencing danger when the thoughts and expectations of others go unrecognized.

Moreover, it is known that the amount of oxytocin modulates different prosocial behaviors (Hollander et al., 2003), such as support to the partner (Grewen et al., 2005) and trust in others (Damasio, 2005). Due to this evidence, an indirect support for the contribution of attachment in hypnosis comes from the key role of oxytocin, which is involved in attachment processes (Galbally et al., 2011; Bosch and Young, 2018; Erkoreka et al., 2022), as well as in hypnotic interaction (Varga and Kekecs, 2014) and susceptibility (Bryant et al., 2012; Bryant and Hung, 2013). However, findings in this field were not always consistent as oxytocin also presented detrimental effects on hypnosis (Declerck et al., 2010; Parris et al., 2014). Moreover, the direct influence of oxytocin in hypnosis cannot be clearly supported since the key-role of the hypnotist-hypnotized relationship and the contribution of other neuromediators need to be considered as well (Zelinka et al., 2014).

The association between attachment and hypnotizability was directly tested by Peter et al. (2011) who, contrary to expectations, observed higher hypnotic susceptibility among insecurely attached individuals. The results however were not replicated by Staudacher et al. (2012) who did not find any association between hypnotizability and attachment styles. Both studies correlated the hypnotizability score provided by the Harvard Group Scale of Hypnotic Susceptibility (HGSHS:A; Shor and Orne, 1963) with the four attachment measures derived from the Relationship Style Questionnaire (RSQ; Griffin and Bartholomew, 2012) It is important to underscore that both studies considered behavioral measures of assessment, while the most recent trends in literature seem to indicate the importance of including phenomenological investigations for understanding hypnosis (Jensen et al., 2017).

Because of the poor literature focusing on this topic, it seems crucial to clarify the role of attachment style in hypnosis as it may help clarify differences in hypnotic abilities, especially when subjectively assessed. To this aim, we carried out two experiments in which hypnosis was assessed through different instruments adopting both behavioral and phenomenological approaches. Findings from these investigations could contribute to understanding interpersonal differences in the ability to grasp hypnosis with relevant implications for both experimental and clinical hypnosis.

## 2 Study 1

### 2.1 Introduction

The present study aimed to investigate the relationship between adult attachment style and hypnotizability, attempting to partially replicate the investigation by Peter et al. (2011). For this purpose, hypnotic susceptibility was quantified through the same assessment procedure used by Peter et al. (2011), i.e., the Harvard Group Scale (see below), in a group of subjects whose attachment style was measured through a questionnaire that considered two main relational factors: anxiety and avoidance. Considering only behavioral scores of hypnotizability, Peter et al. (2011) observed higher responsiveness to hypnosis among insecurely attached subjects. However, results were not confirmed by Staudacher et al. (2012) and, as also proposed by Varga (2021), we hypothesize an opposite relationship, that is the more secure subjects might demonstrate a greater responsivity to hypnotic suggestions than the insecure ones. This possible association is based on the hypothesis that the secure subjects, who are more likely to feel trust and positive emotions in relationships (Bartholomew and Horowitz, 1991), might also be more easily absorbed by a one-to-one hypnotic experience.

### 2.2 Materials and methods

#### 2.2.1 Participants and procedure

Twenty-six hypnosis naïve healthy volunteers participated in this study (14 females, mean age = 24.5 ± 1.2). They were recruited from the student population at the Niccolò Cusano University. The study protocol was approved by the ethical committee of the IRCCS Santa Lucia Foundation (Prot.CE/2024_029) and was in accordance with the ethical standards of the 1964 Declaration of Helsinki. All participants were informed about the procedure and signed the informed consent. The subjects were invited into group hypnosis sessions and were informed that they would be administered a standard hypnotic induction procedure by the experimenter (live administration), who answered all participants’ questions before the HGSHS:A. Then, participants were invited to a second individual session (from 3 to 7 days after the first one) where the attachment style was assessed through a self-report questionnaire (see below). All participants were invited to not share their hypnosis experience with other students.

#### 2.2.2 Harvard Group Scale of Hypnotic Susceptibility:A

The HGSHS:A (Shor and Orne, 1963) consists of a standard hypnotic procedure assessing hypnotizability in terms of “objective” (i.e., behavioral) scoring. The HGSHS:A includes twelve pass/fail items (suggestions) with increasing difficulty (head dropping, eye closing, hand lowering arm immobilization, blocking of fingers, arm rigidity, moving hands, communication inhibition, hallucination, ocular catalepsy, post-hypnotic suggestion and amnesia). Based on their scores, subjects are usually assigned to one of three levels of hypnotic suggestibility, low (0–4), medium (5–8) and high (9–12). It has been validated for the Italian population (De Pascalis et al., 2000) for which a subjective scoring of susceptibility was provided as well: it consists in asking the subjects to evaluate the experience of hypnotic suggestions [e.g., “*with regard to the two suggestions (lowering of the left hand and movement of the hands towards each other), did you have in each of these experiences the subjective conviction that the effect occurred entirely on its own? did you never in any way have the feeling that you were facilitating it?*”].

#### 2.2.3 The Experiences in Close Relationship-Revised Questionnaire

The Experiences in Close Relationship-Revised (ECR-R) Questionnaire is a self-report instrument for the assessment of adult attachment (Fraley and Shaver, 2000) It consists of 18 items on a seven-point Likert scale assessing relational factors with romantic partners: Avoidance and Anxiety. The combination of the factors, returns four attachment styles: secure, avoidant, anxious and preoccupied. The Italian version of the questionnaire was validated by Picardi et al. (2000).

#### 2.2.4 Statistical analysis

Statistical analyses were carried out through Bayesian methods (JASP 0.18.1 software), gaining prominence in the fields of social and behavioral sciences (van de Schoot et al., 2014). One advantage of Bayesian statistic is its capacity to quantify endorsement for study hypotheses, rather than delivering a binary decision on rejecting the null hypothesis. Moreover, the utilization of Bayesian analyses sought to tackle issues linked to a restricted sample size: in fact, they prevent the risks associated with type I or type II errors with multiple comparisons (Hoijtink et al., 2019). Bayesian analyses yield Bayes factors (BF), gauging the extent to which the data favor one hypothesis over another.

Analysis results were interpreted according to Lee and Wagenmakers (2014) and Dienes (2016) whereby evidence for H1 are null (BF = 1), moderate (BF 3 < 10), strong (BF 10 < 30) or very strong (BF 30 < 100).

Correlational Bayesian analyses (Pearson’s r) were performed between the two ECR-R dimensions (anxiety and avoidance) and the two hypnotizability indices (objective and subjective) returned by the HGSHS:A. The overall α level was fixed at 0.05.

### 2.3 Results

Bayesian analysis showed no significant correlations between the ECR-R and the HGSHS:A data (two-sided alternative hypothesis). In particular, anxiety was not associated neither with objective (*r* = −0.39, BF_10_ = 1.21) nor with subjective (*r* = −0.34, BF_10_ = 0.84) hypnotizability. At the same time, avoidance was not associated neither with objective (*r* = 0.21, BF_10_ = 0.40) nor with subjective (*r* = 0.03, BF_10_ = 0.27) hypnotizability.

### 2.4 Discussion

The present study revealed no associations between the behavioral measures of hypnotizability and the patterns of adult attachment, corroborating the Staudacher et al. (2012) results. On the contrary, present findings do not support the observations by Peter et al. (2011) whose contrasting results could be accounted by the different sample sizes, order of test administration or the measurements of attachment: whereas Peter et al. (2011) used the Relationship Scale Questionnaire (RSQ; Griffin and Bartholomew, 2012), we adopted the ECR-R. Also, unlike Peter et al. (2011), we provided live hypnosis: however, administration procedures are unlikely to explain the results as several investigations have demonstrated no differences in hypnotizability between recorded and live hypnosis (see Lush et al., 2021) which on the contrary could have a greater impact in clinical settings.

According to different studies that highlight the limits of an exclusively behavioral assessment of hypnotic susceptibility (Facco, 2022; Perri, 2022; Timmermann et al., 2023), it may be necessary to deepen the hypnotizability through a phenomenological assessment: in such a way, it would be possible to clarify whether attachment style can affect specific dimensions of the interior experience of hypnosis. The hypothesis is in fact that attachment style does not affect hypnotizability (here referred to in its traditional meaning, that is the behavioral responsivity to suggestions), but rather the feeling of hypnosis. Bayesian correlations did not provide evidence in favor of the null or alternative hypothesis, therefore, future studies recruiting larger sample sizes are needed to deepen the possible role of attachment in the different factors of hypnotizability (Woody et al., 2005) as well as in the different subtypes of highly suggestible individuals (Terhune et al., 2011).

## 3 Study 2

### 3.1 Introduction

Growing literature suggests the importance of including subjective measures when assessing hypnotizability (Lush et al., 2018; Facco, 2022; Kekecs et al., 2022; Perri, 2022; Perri and Di Filippo, 2023a,b). In fact, while behavioral methods have historically been the most used, there is now recognition that phenomenological investigations also play a key role in understanding hypnotizability as a multifactorial construct. On the contrary, neglecting the phenomenological variations of consciousness could limit our understanding of the unique experience individuals have during hypnosis (Pekala et al., 2010a). Consequently, in the second study we aimed to deepen the possible relationship between attachment and hypnosis by adopting a retrospective phenomenological assessment (RPA) which quantifies various dimensions of subjective experience during hypnosis. We expect to observe variations in specific dimensions of consciousness as an effect of different relational patterns in intimate relationships.

### 3.2 Materials and methods

#### 3.2.1 Participants

Sixty-eight hypnosis naïve healthy volunteers participated in this study (*N* = 52 females, mean age = 28.4 ± 11.3). They were recruited from the student population at the Niccolò Cusano University. The study protocol was approved by the ethical committee of the IRCCS Santa Lucia Foundation (Prot.CE/2024_029) and was in accordance with the ethical standards of the 1964 Declaration of Helsinki. All participants were informed about the procedure and signed the informed consent. All participants were administered the Phenomenological Consciousness Inventory – Hypnotic Assessment Procedure (PCI-HAP; Pekala et al., 2010a,b) and the ECR-R Questionnaire (see study 1 for details).

#### 3.2.2 Phenomenological hypnotic assessment: the PCI-HAP

The live administration of the PCI-HAP included the Pre-Assessment, the hypnotic procedure, a post-assessment phase, and the PCI. The PCI is a questionnaire consisting of 53 items relating to the phenomenological experience felt during hypnosis (Forbes and Pekala, 1993; Pekala and Kumar, 2000; Pekala et al., 2006, 2010a,b). Each item consists of two dipole items separated by a 7-point Likert scale. The questionnaire is completed retrospectively after hypnotic deinduction.

The PCI-HAP returns different measures such as the self-reported hypnotic depth (srHD) and hypnoidal state score (HSS), the latter of which correlates approximately 0.60 (Forbes and Pekala, 1993) with scores on the Harvard Group Scale of Hypnotic Susceptibility (Shor et al., 1962). The PCI-HAP also explores the phenomenological experience of the hypnotized subject across 14 minor (Joy, Sexual Excitement, Love, Anger, Sadness, Fear, Body image, Time sense, Perception Meaning, Imagery amount, Imagery vividness, Direction of attention, and Absorption) and 12 major dimensions of consciousness (Self-awareness, Altered state, Internal dialogue, Rationality, Volitional control, Memory, Arousal, Positive affect, Negative affect, Altered experience, Imagery, Attention).

#### 3.2.3 Statistical analysis

Statistical analyses were carried out through Bayesian methods (JASP 0.18.1 software; see Study 1 for details): correlational Bayesian analyses (Pearson’s *r*) were performed between the two dimensions of ECR-R (anxiety and avoidance) and the main measures of the PCI-HAP, as well as with the PCI sub-dimensions.

Additionally, according to Italian normative data of the ECR-R (Picardi et al., 2000), participants were categorized into two samples based on their attachment style: secure (66% of the sample) and insecure (34%), which included avoidant, fearful-avoidant, and preoccupied styles. Therefore, a Bayesian Independent Sample *t*-test was performed between the two groups (secure vs. insecure) for the PCI sub-dimensions. The overall α level was fixed at 0.05. According to Lee and Wagenmakers (2014) and Dienes (2016) whereby evidence for H1 are null (BF = 1), moderate (BF 3 < 10), strong (BF 10 < 30) or very strong (BF 30 < 100).

### 3.3 Results

The Bayesian correlation showed no significance between the ECR-R dimensions (anxiety and avoidance) and the sr-HDS and HSS score. As for the PCI sub-dimensions, a strong positive correlation emerged between the ECR-R anxiety and the internal dialogue (*r* = 0.35, BF_10_ = 19): the data were 19 times more likely under the alternative than the null hypothesis: anxious subjects revealed higher levels of internal dialogue. Also, a moderate positive correlation emerged between the avoidance factor of the ECR-R with anger (*r* = 0.31, BF_10_ = 7.5), sadness (*r* = 0.32, BF_10_ = 8.8) and negative affect (*r* = 0.31, BF_10_ = 7.1): anxious subjects revealed higher level of anger, sadness and negative affect. Finally, a moderate negative correlation emerged with absorption (*r* = −0.26, BF_10_ = 3): more avoidant subjects experience lower absorption. See Table 1 for a summary of the Bayesian correlational analysis.

**Table 1:** The Bayesian correlations (Pearson’s *r*) between the ECR-R factors (avoidance and anxiety) and the sub-dimensions of the PCI.

		Pearson’s *r*	BF_10_
Anxiety	Internal dialogue	0.35	19.04
Avoidance	Anger	0.31	7.54
	Sadness	0.32	8.82
	Negative affect	0.31	7.07
	Absorption	−0.26	3.00

**Figure 1:**
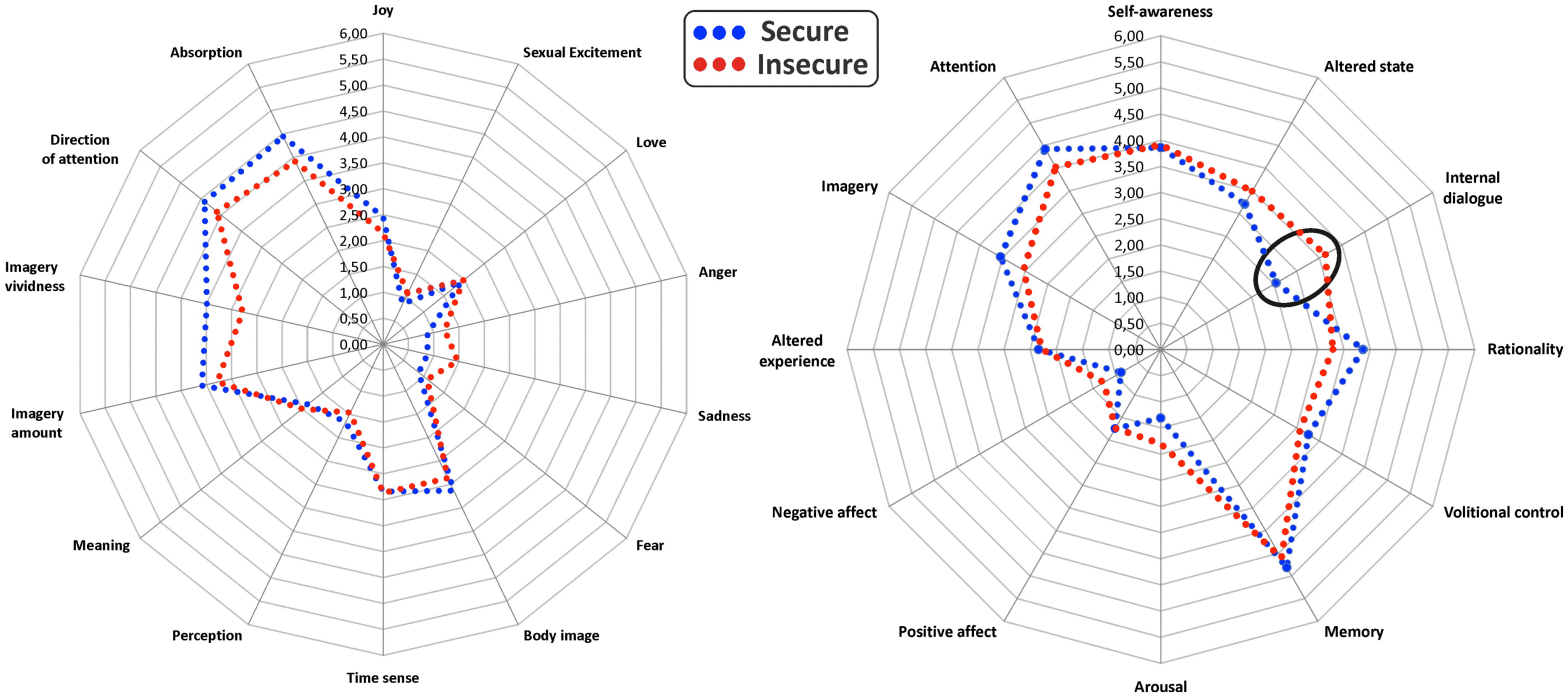
Radar of the minor (left) and major (right) dimensions of consciousness as identified by the PCI. The scores for the secure and insecure attachment groups are shown.

The Bayesian independent t-test showed a significant difference for the PCI subdimension of internal dialogue. A two-sided analysis between the secure and insecure revealed a Bayes factor (BF_10_) showing that the data were 11.06 times more likely under the alternative than the null hypothesis: insecure subjects revealed higher levels of internal dialogue. Figure 1 shows the major and minor PCI dimensions for the two groups.

### 3.4 Discussion

In line with our hypothesis, the study revealed an association between attachment patterns and certain variations of consciousness during hypnosis. In fact, the greater the anxiety pattern of attachment the greater the internal dialogue during hypnosis. Furthermore, the avoidance pattern of attachment affected the interior experience of hypnosis, especially in terms of greater sadness, anger, negative affect and conversely, reduced absorption. The latter is defined as “*an extreme involvement with one object, idea, or pursuit, with inattention to other aspects of the environment*” (VandenBos, 2007) and is typically identified as a feature of the hypnotic experience (Facco et al., 2017). Presumably, repeated failures in the intimate relationships negatively affected the individuals’ ability to be focused on hypnosis due to their difficulty placing trust in others. The group comparisons (secure-vs. insecure-attached) confirms the tendency of insecure subjects to experience more internal dialogue during hypnosis: in other words, insecure attachment could impair the ability to turn attention away from the surroundings because of the need to be in control, or the tendency to maintain a low emotional intensity to disengage from involving relations. In fact, internal dialogue is intended as the self-talk of participants during hypnosis. A high internal dialogue can be a critical aspect of hypnosis as the subject may find it difficult to focus on the therapist voice due to his/her thoughts.

## 4 General discussion

Hypnosis is a complex field and people’s responses depend on many factors, including personality (Gibson and Corcoran, 1975), beliefs (Phillips et al., 2022) empathy (Barrett, 2016) and more. The attachment style-hypnosis relationship has been proposed as well (Varga, 2021), although this connection has received very little attention in experimental hypnosis. The aim of the present research was to test whether adult attachment styles could influence how an individual responds to hypnotic procedures.

*‘Although particularly evident in early childhood, attachment behavior characterizes human beings from cradle to grave*’ (Bowlby, 1991): this sentence reflects the role of attachment in influencing how individuals interpret their life experiences. For this reason, we decided to investigate the hypnosis-attachment relationship by considering both “objective” and subjective measurements. To the best of our knowledge, only three studies tested the role of attachment on hypnotizability: all investigations adopted behavioral measures to quantify the hypnotic susceptibility (Peter et al., 2011, 2014; Staudacher et al., 2012) obtaining mixed results on the role of insecure attachment. On the other hand, Varga (2021) hypothesized that the more secure subjects might show greater responsiveness to hypnotic than insecure ones.

In Study 1, in contrast to Peter et al. (2011), we found no significant association between attachment styles and the objective measure of hypnotic susceptibility as returned by the HGSHS:A (Shor and Orne, 1963). This result may be explained by the sample size, but also by the different adult attachment tests. To directly explore the variations of consciousness, a phenomenological measurement of hypnosis was included in Study 2 (i.e., the PCI-HAP) that indicated the insecure attached individuals as experiencing more internal dialogue during hypnosis when compared to secure ones. In other words, subjects who experience discomfort in intimate relationships were less absorbed into the hypnosis experience, also reporting a greater intensity of negative emotions.

Overall, the role of attachment in hypnosis suggests that early parent–child relational patterns play a significant role in shaping an individual’s ability to engage in any kind of intimate relationship, not necessarily affective or sexual. In fact, secure attachment and the development of self-regulation skills were associated with higher absorption during hypnosis, while insecure attachment may contribute to impair the attentional and emotional features of the hypnotic experience.

In summary, the present studies indicate that attachment styles could influence a person’s feeling of hypnosis, and that considering only external behaviors of hypnosis may not fully capture the richness of inner sensations. As a further confirmation, when considering the external signs of hypnotizability (i.e., the objective score of the Harvard test), no relationships emerged with any of the attachment factors (see Results of Study 1). These findings further highlight the need for considering the inter-individual differences as well as the phenomenological perspective when assessing hypnosis and hypnotizability. As for the clinical implications, these studies suggest the hypnotherapist should consider different approaches for attachment styles: while the secure attached appears to be excellent candidates for hypnosis interventions, the insecure ones might need more reassurance and a good therapeutic relationship before engaging in hypnotic procedures. Also, suggestions for calm, absorption, and reduction of self-talk could be provided to prevent the hypnotic patterns of the insecure attached.

## Data availability statement

The raw data supporting the conclusions of this article will be made available by the authors, without undue reservation.

## Ethics statement

The studies involving humans were approved by IRCCS Santa Lucia Foundation (Prot.CE/2024_029). The studies were conducted in accordance with the local legislation and institutional requirements. The participants provided their written informed consent to participate in this study. Written informed consent was obtained from the individual(s) for the publication of any potentially identifiable images or data included in this article.

## Author contributions

GDF: Conceptualization, Formal analysis, Investigation, Writing – original draft. RLP: Conceptualization, Supervision, Writing – review & editing.
